# Distinct neuronal processes in the ventromedial prefrontal cortex mediate changes in attention load and nicotine pro-cognitive effects in male rats

**DOI:** 10.3389/fncir.2025.1540975

**Published:** 2025-03-07

**Authors:** Caroline Vouillac-Mendoza, Nathalie Biendon, Sandra Dovero, Karine Guillem

**Affiliations:** ^1^Univ. Bordeaux, CNRS, INCIA, UMR 5287, Bordeaux, France; ^2^Univ. Bordeaux, CNRS, HistoCARE Facility, IMN, UMR 5293, Bordeaux, France

**Keywords:** attention, prefrontal cortex, neuronal processes, electrophysiology, nicotine

## Abstract

The prefrontal cortex (PFC) plays a key role in attention. In particular, neuronal activity in the ventromedial PFC (vmPFC) has been implicated in the preparatory attentional period that immediately precedes cue presentation. However, whether vmPFC neuronal activity during this preparatory period is also sensitive to changes in task demand and to the pro-cognitive effects of nicotine remained to be investigated. Here, we used *in vivo* electrophysiology to record vmPFC neuronal activity in rats during two distinct manipulations: a task manipulation that increased task demand by reducing the cue stimulus duration (from 1 to 0.5 s), and a pharmacological manipulation by administrating an acute nicotine injection (10 μg/inj, i.v.) before the session. We found that increasing task demand decreased attentional performances and vmPFC precue neuronal activity, but had no effect on gamma oscillations. In contrast, nicotine injection increased attention and gamma oscillations, but almost abolished vmPFC phasic precue responses. Together, these findings indicate the existence of two distinct neuronal processes operating at different timescales and suggests that allocation of attention could be achieved through multiple neuronal mechanisms within the vmPFC.

## Introduction

The prefrontal cortex (PFC) plays a critical role in attentional processes in humans and animals (Miller and Cohen, 2001; Christakou et al., 2004; Totah et al., 2009; Kim et al., 2016), and dysfunctions of the PFC have been implicated in several disorders, including attentional deficit hyperactivity disorder (ADHD), Alzheimer’s disease or schizophrenia (Dalley et al., 2004; Chudasama and Robbins, 2006; Halperin and Schulz, 2006). In rodents, PFC-dependent cognitive functions have been extensively studied using the 5-choice serial reaction time task (5-CSRTT), a well-established test setup that taxes various aspects of attentional control over performance (Robbins, 2002; Bari et al., 2008).

Previous electrophysiologic recordings in both primates and rodents have implicated mPFC activity during attention tasks (Niki and Watanabe, 1979; Gill et al., 2000; Johnston et al., 2007; Terra et al., 2020). Specifically, these studies have demonstrated that attention-related mPFC neuronal activity typically occurs in anticipation of oncoming task-relevant visual cues on a time scale of seconds (Totah et al., 2009; Totah et al., 2013; Donnelly et al., 2015; Kim et al., 2016). In particular, the ventromedial mPFC (vmPFC) neuronal activity plays an important role during the seconds that immediately precede cue presentation (Luchicchi et al., 2016). Moreover, gamma oscillations in the mPFC also play a key role in attentional processing in humans and animals (Fries et al., 2001; Womelsdorf and Fries, 2007; Kim et al., 2016; Bueno-Junior et al., 2017) and increased gamma oscillations improve attention (Kim et al., 2016; Bueno-Junior et al., 2017).

Several factors or task manipulations have been shown to alter attentional behavior. For instance, increasing task demand by reducing the stimulus duration during a 5-CSRTT session decreased attention, while in contrast nicotine administration before the session increased it (Mirza and Stolerman, 1998; Hahn et al., 2002; Robbins, 2002; Amitai and Markou, 2009). One open question, however, is whether vmPFC neuronal activity is sensitive to changes in task demand and to the pro-cognitive effects of nicotine. Here, we used *in vivo* electrophysiological recordings in rats trained to perform an attentional 5-CSRTT task to directly assess this question. We used two distinct manipulations: a task manipulation that increased task demand by reducing the cue stimulus duration (from 1 s to 0.5 s), and a pharmacological manipulation by administrating an acute injection of nicotine (10 μg/inj, i.v.) before the session. We then recorded and compared *in vivo* vmPFC neuronal activity during these two manipulations.

## Materials and methods

### Subjects

A total of 28 adult male Wistar rats (250–275 g at the beginning of experiments, Charles River, Lyon, France) were used (8 for the *in vivo* electrophysiology experiment, and 20 for the optogenetic experiment). Rats were housed in groups of 2 and were maintained in a light- (reverse light–dark cycle), humidity- (60 ± 20%) and temperature-controlled vivarium (21 ± 2°C), with water available ad libitum. Animals were food restricted throughout the experiment to maintain at least 95% of their free feeding body weight. Food ration (~14–16 g/rat) were given 3 h after the end of the session. All behavioral testing occurred during the dark phase of the light–dark cycle. Home cages were enriched with a nylon gnawing bone and a cardboard tunnel (Plexx BV, The Netherlands). All experiments were carried out in accordance with institutional and international standards of care and use of laboratory animals [UK Animals (Scientific Procedures) Act, 1986; and associated guidelines; the European Communities Council Directive (2010/63/UE, 22 September 2010) and the French Directives concerning the use of laboratory animals (décret 2013–118, 1 February 2013)]. The animal facility has been approved by the Committee of the Veterinary Services Gironde, agreement number B33-063-922.

### Five-choice serial reaction time task (5-CSRTT)

#### Apparatus

**Figure 1:**
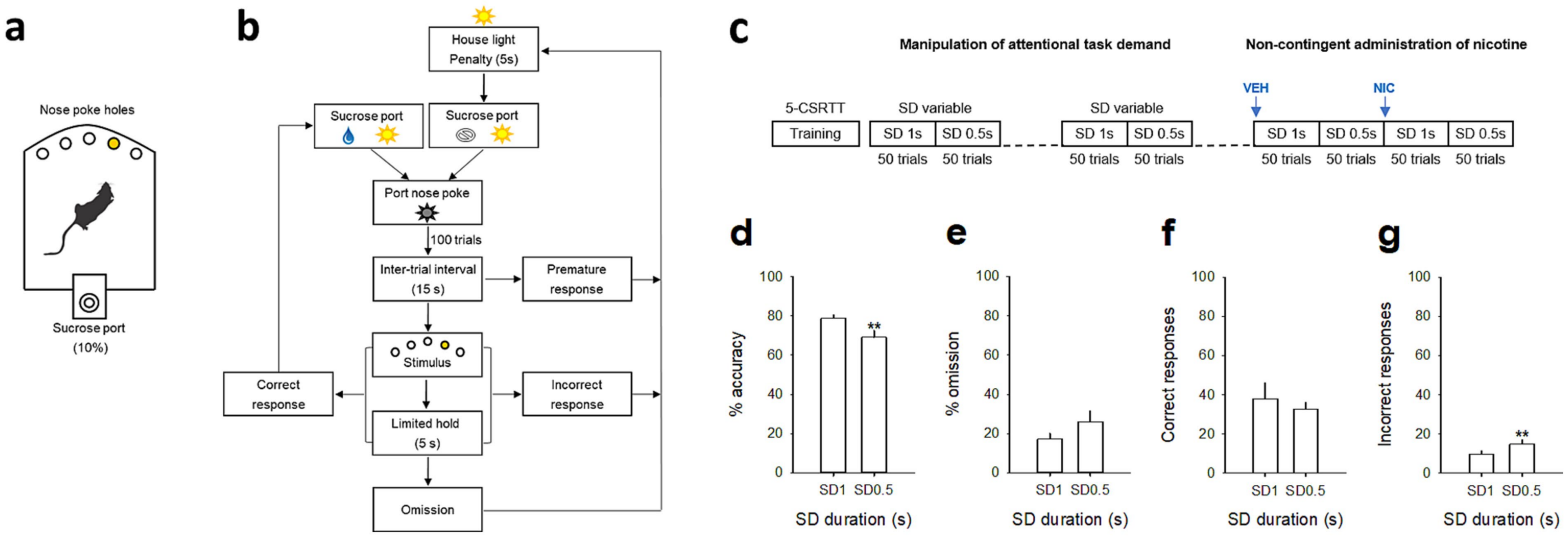
Attentional performances in 5-CSRTT during a stimulus duration (SD) variable procedure. **(a)** Schematic of the 5-CSRTT apparatus and **(b)** diagram showing the sequence of events during a 5-CSRTT training session. Rats initiate trials by responding into the sucrose port; after an intertrial interval delay, a brief stimulus is presented in one of the five holes. Subjects must respond to that hole (correct response) within a certain time (limited hold) to receive a sucrose reward (0.1 mL of 10% sucrose). If they respond to the wrong hole (incorrect response), respond before the stimulus is presented (premature response), or fail to respond (omission), they are punished with a penalty period during which the house light was turned on. After that, the sucrose port is switched on and rats have to respond into it to initiate the next trial. **(c)** Timeline of the experiments. Animals were first trained under 5-CSRTT sessions then under a SD variable procedure (50 trials at SD 1 s followed by 50 trials at SD 0.5 s). Recordings were performed during the last SD variable session, and after a non-contingent saline (VEH) and nicotine administration (NIC, 10 μg/inj, i.v.), each administered 1 min before a SD variable session. **(d,g)** Mean (±SEM) percentage of accuracy **(d)**, omission **(e)** and **(f,g)** mean (±SEM) number of correct **(f)** and incorrect **(g)** responses as a function of the light cue stimulus duration, respectively SD 1 s and SD 0.5 s. **p < 0.01, different from SD 1 s.

Height identical five-hole nose poke operant chambers (30 cm × 40 cm × 36 cm) housed in sound-insulating and ventilated cubicles were used for 5-CSRTT testing and training (Imétronic, Pessac, France) (Figure 1a), as previously described (Vouillac-Mendoza et al., 2024). Each chamber was equipped with stainless steel grid floors and a white house light mounted in the center of the roof. Set in the curved wall of each box was an array of five circular holes (2.5 cm sides, 4 cm deep and positioned 2 cm above the grid floor) each equipped with internal light-emitting diodes and an infrared sensor detecting the insertion of the animals’ nose. The opposite wall was not curve and equipped with a delivery port and a drinking cup mounted on the midline. The delivery port was illuminated with a white light diode mounted 8.5 cm above the drinking cup. A lickometer circuit allowed monitoring and recording of licking. Each chamber was also equipped with a syringe pump placed outside, on the top of the cubicle, which delivered sucrose solution into the drinking cup through a silastic tubing (Dow Corning Corporation, Michigan, United States).

#### General training procedure in the 5-CSRTT

During habituation, rats (*n* = 28) were exposed 60 min to the boxes for two sessions, with the white noise on. During the next three sessions, animals were trained to associate the delivery port with reward without any response requirement. Sucrose rewards (0.1 mL of 10% sucrose) were delivered into the drinking cup with a variable interval of 15 s and were signaled by the illumination of the drinking port. Each session terminated after a maximum of 100 sucrose rewards or 60 min, whichever came first. In the next sessions, all five holes were illuminated and rats had to nose pokes into one of them to obtain a sucrose reward as above. Then, only one hole was illuminated and animals were trained to nose poke into that particular hole to obtain a sucrose reward as above.

In the next 5-CSRTT sessions, rats were trained with a maximum number of 100 consecutive self-paced trials per session or a maximum duration of 60 min whichever came first (Figure 1b). The opportunity to self-initiate a trial was signaled by turning on the white light diode in the delivery port. To initiate a trial, rats had to turn off the illuminated port by visiting it (i.e., exit after entering) within 1 min. If no visit occurred within the imparted time, the port was turned off automatically and this marked trial onset. After a fixed preparation time of 15 s, a brief light stimulus was presented behind one of the 5 holes on the opposite curved wall (pseudorandom selection across trials). To receive a reward (0.1 mL of 10% sucrose), animals had to nose-poke the illuminated hole either during the stimulus presentation or within a post-stimulus limited hold period of 5 s (Figure 1b). Correct nose-poke responses immediately turned back on the light in the delivery port and triggered the delivery of sucrose into the drinking cup. Incorrect nose-poke responses in one of the dark holes were not rewarded by sucrose, but were punished by a time-out penalty of 5 s signaled by turning on the house light. If animals failed to respond in any of the holes during a trial, this was considered an omission response. Omissions, like incorrect responses, were punished by a signaled 5-s time-out penalty. At the end of the time-out penalty, the house light was switched off and the light in the delivery port was turned back on for the next trial. During training, the duration of light stimulus was initially set to 30 s and progressively decreased across sessions to 1 s until the subject met performance criteria (omissions <30%; accuracy >60%; number of self-initiated trials >50). All animals meet these criteria and were next trained for an additional 10 SD 1 s sessions until stabilization of their performances.

#### Manipulation of attentional task demand

Next, attentional demand was further increased within-session by decreasing the duration of the light stimulus (i.e., stimulus duration, SD) in the following order: 1 and 0.5 s (SD 1 s and SD 0.5 s). Each stimulus duration (SD) was tested during 50 trials per session (50 trials at SD 1 s followed by 50 trials at SD 0.5 s) so that animals experienced the same number of trials at each SD (Figure 1c). Importantly, although the order of the two SD were not counterbalanced, we have previously shown that the degree of motivation, or satiety remained stable across the entire session and could thus not account for changes in behavioral performances between the two SD (Vouillac-Mendoza et al., 2024). Moreover, animals were oriented toward the curved wall and were engaged in the task in the majority of the trials (Vouillac-Mendoza et al., 2024). Animals were tested in this SD variable procedure during several daily sessions until stabilization of performance. Each session terminated after a maximum of 100 sucrose rewards or 60 min, whichever came first.

#### Non-contingent administration of nicotine

Finally, we assessed the effect of a non-contingent administration of nicotine on attentional performance. A catheter was implanted in the right jugular vein (see below *Surgeries* for details) and a saline (VEH) or a nicotine injection (NIC, 10 μg/injection, i.v.) was administered intravenously through the jugular vein 1 min before the SD variable session (Figure 1c). Animals first received an intravenous saline injection before the SD variable session, then a nicotine injection (10 μg/injection, i.v.) before the second SD variable session.

### *In vivo* electrophysiological recordings

#### Surgeries

Animals were anesthetized with a mixture of ketamine (100 mg/kg, i.p., Bayer Pharma, Lyon, France) and xylazine (15 mg/kg, i.p., Merial, Lyon, France) and surgically prepared with an indwelling silastic catheter in the right jugular vein (Dow Corning Corporation, Michigan, United States). Then, under isoflurane anesthesia, an array of 16 teflon-coated stainless steel microwires (2 rows of 8 wires separated from each other by 0.25 mm, MicroProbes Inc., Gaithersburg, MD) were implanted unilaterally in the vmPFC [AP: + 2.5 to +4.5 mm, ML: 0.3 to 1.2 mm, and DV: −4.5 mm relative to skull level], as previously described (Girardeau et al., 2019; Abarkan et al., 2023). A stainless-steel ground wire was also implanted 4 mm into the ipsilateral side of the brain, 5 mm caudal to bregma. After surgery, catheters were flushed daily with 0.2 mL of a sterile antibiotic solution containing heparinized saline (280 IU/ml) and ampicilline (Panpharma, Fougères, France). Behavioral testing began 10–14 days after surgery.

#### Neuronal and LFPs recordings

Voltage signals from each electrode will be recorded, amplified, bandpass filtered (from 250 Hz to 8 kHz for unit activity and from 0.7 Hz to 170 Hz for local field potentials LFPs) and digitally captured using commercial hardware and software (OmniPlex, Plexon, Inc., Dallas, TX). Neuronal activity was digitized at a rate of 40 kHz and LFPs were down sampled to 1 kHz. Behavioral events in the operant task were streamed to the Plexon via TTL pulses delivered from the Imetronic system (Imetronic, Pessac, France) to allow the neuronal data to be accurately synchronized and aligned to these behavioral events. Single units spike sorting was performed off-line: principal component scores were calculated and plotted in a three-dimensional principal component space and clusters containing similar valid waveforms were defined (Offline Sorter, Plexon). The quality of recorded units was ensured with an interspike interval criterion (>1 ms) and a signal:noise criterion (>3X noise band). Neurons were classified into putative pyramidal and interneurons according to the waveform spike width and average firing rate, as previously described (Guillem et al., 2010; Girardeau et al., 2019). Due to the small number of interneurons (*n* = 13), data analysis was exclusively focused on putative pyramidal cells and consisted of a total 184 pyramidal neurons (123 during the SD variable procedure session and 61 during the non-contingent nicotine test session). We recorded on average 15.6 ± 1.8 neurons per animal. Each animal was recorded during three sessions: (1) after stabilization of the performance under a SD variable procedure (50 trials at SD 1 s followed by 50 trials at SD 0.5 s), and (2) after a non-contingent saline and nicotine administration (10 μg/inj, i.v. through the jugular vein) 1 min before the SD variable session (Figure 1c).

#### Neuronal data analysis

Electrophysiological data were analyzed using NeuroExplorer (Plexon) and Matlab (Mathworks, Natick, MA). Perievent firing rate histograms (PETHs) were used to analyze single cell neuronal responses during the attentional precue period (−2 to 0 s before cue onset) when attention is allocated as previously indicated (Totah et al., 2009; Totah et al., 2013), and compared between the three trials types determined by the subsequent cue hole nose poke response (correct or incorrect) or lack of nose poke response (omission) after the cue onset. To examine population activity, PETHs for each unit were normalized in *z*-score and averaged across different trials (correct, incorrect, and omission), as previously described (Kim et al., 2016). To provide a better visualization of the data, population activity graphs were smoothed with a Gaussian filter. Neurons were tested for phasic changes during the attentional process by comparing firing rates during the precue period (−2 to 0 s before cue onset) to baseline firing rates during −6 to −4 s before cue onset using a Wilcoxon test (*p* < 0.05), as previously described (Totah et al., 2009). The percentage of neurons was determined for each trials type and each SD duration and compared using the two-proportion *z*-test. To test for the acute pharmacological effects of nicotine on overall neuronal activity, we compared firing rate (*z* score) of all recorded neurons during the first 25 min of the 5-CSRTT session after a saline injection to firing during the first 25 min 5-CSRTT session after a nicotine challenge injection (10 μg/inj, i.v. 1 min before the session), using a one-way repeated ANOVA. At the end of the study, histological procedures were used to identify the location of all wire tips used to record neurons. For spectral analysis, the power spectral density (PSD) of each LFP was calculated with the NeuroExplorer PSD function using a multitaper fast Fourier transform (FFT), with a Hamming window of 2048 points (2 s), 50% overlap and 1 Hz resolution, as previously described (Guillem and Ahmed, 2020). PSD values were expressed as the percent of the total power spectrum within the frequency range considered (gamma oscillations 30–80 Hz).

### *In vivo* optogenetic manipulation

#### Surgery and procedures

The effect of vmPFC pyramidal neurons optogenetic inhibition was tested in another group of 20 rats. Under isoflurane anesthesia, light-gated opsins viruses encoding the archaerhodopsin-3.0 protein (*n* = 12; CaMKII-*α*-eArchT3.0-eYFP; 5.5 × 10^12^ viral molecules/mL; UNC Vector Core) or a control virus (*n* = 8; CaMKII-eYFP 4.4 × 10^12^ viral molecules/mL; UNC Vector Core) were infected bilaterally in the vmPFC (AP: +3 mm; ML: ± 1.4 mm; DV: −4.5 from skull, angle 10°) to selectively infect pyramidal neurons (Abarkan et al., 2023). A total of 0.5 μL/hemisphere was injected at a rate of 0.1 μL/min for 10 min using an Hamilton syringe mounted in an infusion pump after which the injector will be left in place for an additional 10 min to allow virus diffusion. Optic fibers (200/230 μm core diameter Plexon Inc., Dallas, TX) were implanted bilaterally slightly above the injection site (~0.3 mm) to ensure illumination of the transduced neurons and secured to the skull screws and dental cement. Seven days after surgery, rats were retrained under the SD variable for 20 additional days before being testing for optogenetic manipulations. Opto-inhibition of vmPFC pyramidal neurons (8–11 mW) during 2 s before the cue presentation (Luchicchi et al., 2016) was assessed in two separate SD variable sessions (No light and Light application, random order). Each implanted optic fiber was connected to a LED module (550 nm) mounted on a dual LED commutator connected to an optogenetic controller (PlexBright, Plexon Inc., Dallas, TX).

#### cFos immunohistochemistry

We used cFos immunohistochemistry to check the efficiency of the opto-inhibition of vmPFC neuronal activity. Animals underwent a challenge SD 1 s session during which vmPFC pyramidal neurons were opto-inhibited as previously. Ninety min after the start of the challenge session, rats were anesthetized and perfused transcardially with 4% paraformaldehyde (PFA). Brains were removed, serially cut on a cryostat (50 μm) and stored in PBS-0.2% sodium azide as free-floating sections for immunohistochemistry. Brains were first checked for fiber placement and viral expression. Some floating sections were put in a blocking solution with Normal Donkey Serum 5% in PBS-Triton 0.3% (PBST) during 90 min and then incubated with rabbit anti-GFP antibody (1:2000, Invitrogen, A11122) and mouse anti-CAMKII antibody (1:500, Thermo Fisher, MA1-048) overnight at room temperature (RT). Sections were next washed with PBST and revealed with donkey anti-rabbit antibody conjugated to Alexa 488 (1: 1000, Invitrogen, A21206) and donkey anti-mouse antibody conjugated to A568 (1: 1000, Invitrogen, A10037) for 90 min at RT. Sections were finally mounted in Vectashield medium (Vector Laboratories), coverslipped, imaged on an epifluorescence microscope (Olympus) and analyzed using ImageJ (NIH, United States). Others floating sections were processed for cFos immunohistochemistry analysis as described in details elsewhere (Mihindou et al., 2013; Navailles et al., 2015). Briefly, floating sections were first blocked for unspecific staining in 5%BSA-0.3% triton X100 PBS solution and then incubated over night at RT with rabbit polyclonal anti-cFos antibody (1: 1000; sc-52, Santa-Cruz Biotechnology) diluted in 1%BSA-0.3%Triton-X100 0.01 M PBS. On the next day, sections were rinsed in PBS and incubated 30 min in an anti-rabbit polymer system (Anti-rabbit Envision HRP polymer system, Agilent K400311-2). After several rinses, sections were incubated in DAB solution (Envision DAB kit: Agilent K346811-2) to reveal the cFos positive cells. Sections were finally mounted on gelatined coated slides, coverslipped, scanned with a Panoramic Scanner (3D-Histech, Hungary) and analyzed using the Mercator software (Immascope, France). Density of cFos-expressing cells (cells/mm^2^) was measured by a blind observer in both hemispheres of each rat and averaged.

### Drugs

Nicotine hydrogen tartrate was purchased from Sigma (St. Louis, MO), dissolved in isotonic NaCl (0.9% w/w saline in water), filtered through a syringe filter (0.22 μm) and stored at room temperature. Drug dose was expressed as free base.

### Data analysis

The following behavioral measures were used for analysis: % accuracy ([100 × correct responses]/[correct responses + incorrect responses]); % omission (100 × number of omissions/total self-initiated trials); premature responses (number of responses that occurred before the presentation of the light stimulus); latency of correct and incorrect responses; and, finally, reward latency (i.e., latency between correct responses and contact with the drinking cup). Behavioral data were subjected to one-way or two-way ANOVAs with SD duration (SD 1 s or SD 0.5 s) and nicotine treatment (VEH or NIC) as repeated measures, followed by Tukey *post hoc* tests where relevant. Electrophysiological study, data were subjected to two-way ANOVAs with trial type (correct, incorrect or omission) as between factor and time as repeated measures, followed by Tukey *post hoc* tests where relevant. Percentage of neurons were compared using the two-proportion *z*-test. Optogenetic study, data were subjected to two-way ANOVAs with virus type (ArChT or YFP) as between factor and light condition (light or no-light) as repeated measures, followed by Tukey *post hoc* tests where relevant. Statistical analyses were run using Statistica, version 7.1 (Statsoft Inc., Maisons-Alfort, France).

## Results

### Attentional performances vary with attentional load

We first determined animals’ attentional capacities using the five-choice serial reaction time task (5-CSRTT). Briefly, animals (*n* = 8) were trained to detect and respond to a brief light stimulus randomly presented in one of five nose poke holes to receive a sucrose reward (0.1 mL of 10% sucrose), as previously described (Vouillac-Mendoza et al., 2024) (see Materials and methods for details; Figures 1a,b). During the training schedule, the stimulus duration (SD) progressively decreased across sessions until they reached stable performance with a final SD 1 s (from 30s to 1 s, reached after 36 ± 2 training sessions). To further test attention, attentional load was then increased using a SD variable procedure, in which stimulus durations decreased from 1 to 0.5 s within the same session (Figure 1c). Animals were trained under this SD variable procedure for 10 more days until they reached stable performance. As expected, reducing the SD duration from 1 s to 0.5 s impaired the attentional performances of the rats (*n* = 8) and resulted in a significant decrease in accuracy [from 78.7 ± 2.0% to 69.1 ± 3.4% for SD 1 s and SD 0.5 s respectively; *F*(1,7) = 11.23; *p* = 0. 0097; Figure 1d], and a small but not significant increase in the percentage of omission [*F*(1,7) = 3.91; *p* = 0.08; Figure 1e]. The decrease in accuracy during SD 0.5 s was mainly caused by an increase in incorrect responses [*F*(1,7) = 12.81; *p* = 0.0089; Figure 1g], rather than a decrease in correct responses [*F*(1,7) = 1.14; *p* = 0.32; Figure 1f]. Furthermore, there was no effect of SD duration on any other all behavioral measures, such as number of premature responses [*F*(1,7) = 1.8; *p* = 0.21], correct response latency [*F*(1,7) = 2.6; *p* = 0.15], incorrect response latency [*F*(1,7) = 3.8; *p* = 0.09], or latency to collect sucrose reward [*F*(1,7) = 0.4; *p* = 0.52; Table 1], suggesting that decreased accuracy reflected impairments in attention processes rather than motor or motivational deficits.

**Table 1:** 5-CSRTT performances at varying stimulus durations.

	Stimulus durations (s)	
	SD 1 s	SD 0.5 s
Premature	7.8 ± 2.5	5.5 ± 1.4
Correct latency (s)	1.1 ± 0.0	1.0 ± 0.1
Incorrect latency (s)	1.4 ± 0.1	1.2 ± 0.1
Reward latency (s)	1.5 ± 0.1	1.6 ± 0.2

### vmPFC neuronal phasic activity is associated with changes in attentional performances

**Figure 2:**
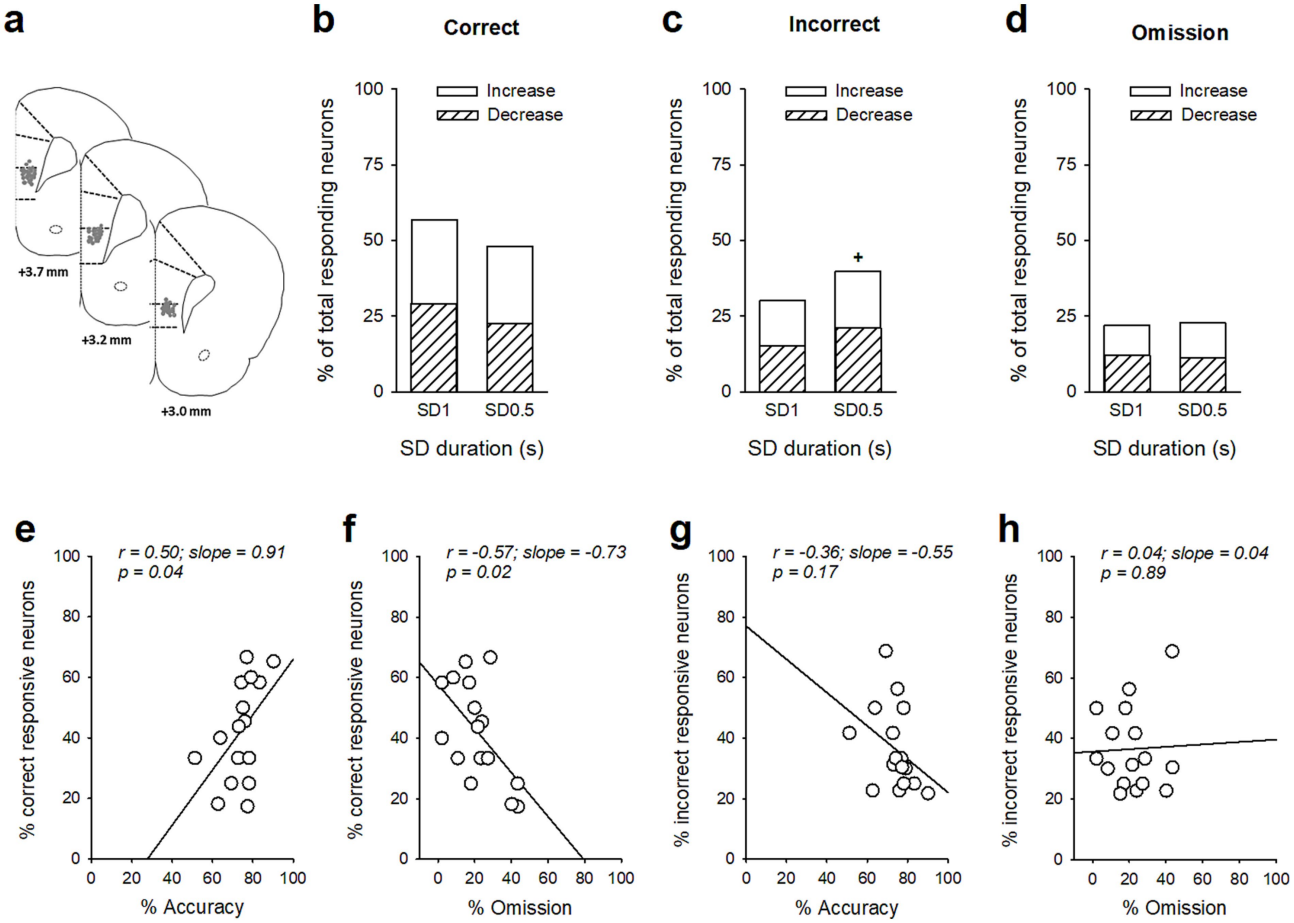
Ventromedial PFC (vmPFC) precue phasic responses during the SD variable procedure. **(a)** Schematic of the location of individual wire tips within the vmPFC. The numbers indicate millimeters anterior to bregma. **(b–d)** Percentage of total neurons responding before the cue on correct **(b)**, incorrect **(c)**, and omission trials **(d)** as a function of the SD duration, respectively SD 1 s and SD 0.5 s. Excitatory units are shown as solid bars, and inhibitory units are shown as hashed bars. ^+^*p* < 0.05, different from SD 1 s. **(e,f)** The individual % of responsive neurons on correct trials was positively correlated with individual % of accuracy **(e)** and negatively correlated with individual % of omission **(f)**, **(g,h)** but the % of responsive neurons on incorrect trials was not correlated with the % of accuracy **(g)** or omission **(h)**.

We next recorded *in vivo* vmPFC neuronal activity during this SD variable procedure in the same animals (Figure 2a). Based on previous reports (Totah et al., 2009; Totah et al., 2013; Kim et al., 2016), we focused our examination on the responses of vmPFC pyramidal neurons recorded during the precue period (−2 to 0 s before cue onset), when attention is allocated. vmPFC neurons whose firing activity changed during the precue period were identified for each SD duration, and neuronal responses were divided into three groups depending on whether a correct response, an incorrect response, or an omission of response followed. Precue-related phasic responses were observed on each trial and consisted of mixed changes in activity during the precue period with half of the neurons showing an increase and half of the neurons showing a decrease in firing activity on every trials type and SD durations. However, the proportion of phasic precue responses were different depending on trial type and SD duration. Table 2 lists the number and percentage of precue responsive neurons on each trial type (correct, incorrect and omission trials) and for each SD duration (SD 1 s or SD 0.5 s). Notably for the SD 1 s with high attentional performances, the proportion of precue responsive neurons was higher on correct trials than on incorrect (57% vs. 30%, *Z* = 4.24; *p* < 0.001; Figures 2b,c and Table 2) or omissions trials (57% vs. 22%, *Z* = 5.61; *p* < 0.001; Figures 2b,d and Table 2). For the shorter SD 0.5 s with low attentional performances in contrast, the proportion of precue responsive neurons was similar on correct and incorrect trials (48% vs. 40%, *Z* = 1.29; NS Figures 2b,c and Table 2), while still higher than on omission trials (48% vs. 23%, *Z* = 4.28; *p* < 0.001; Figures 2b,d and Table 2). The weakened difference between the proportion of correct and incorrect precue responsive neurons observed when the SD decrease to 0.5 s was mainly caused by an increased responding during incorrect trials (30% vs. 40%, *Z* = 1.60; *p* < 0.05) rather than a decreased responding during correct trials (57% vs. 48%, *Z* = 1.40; *p* = 0.08). Moreover, the percentage of responsive neurons on correct trials was positively correlated with the percentage of accuracy (*r* = 0.50; slope = 0.91; *p* = 0.049) and negatively correlated with the percentage of omission (*r* = −0.57; slope = −0.73; *p* = 0.02), while no correlations were observed with the percentage of responsive neurons on incorrect trials (*r* = −0.36; slope = −0.55 and *r* = 0.04; slope = 0.040; *NS*, for accuracy and omission, respectively).

**Table 2:** Number and percentage of vmPFC neurons responding during the precue period.

	Correct trials		Incorrect trials		Omission trials	
	SD 1 s	SD 0.5 s	SD 1 s	SD 0.5 s	SD 1 s	SD 0.5 s
Total	70 (60%)	59 (48%)	37 (30%)***	49 (40%)^+^	27 (22%)***	28 (23%)***
Increase	34 (27%)	31 (25%)	18 (15%)**	23 (19%)	18 (15%)**	14 (11%)**
Decrease	36 (29%)	28 (23%)	19 (15%)**	26 (21%)	19 (15%)**	14 (11%)**

***p < 0.001 and **p < 0.01, significant difference compared to correct trials. ^+^*p* < 0.05, significant difference compared to SD 1 s.

**Figure 3:**
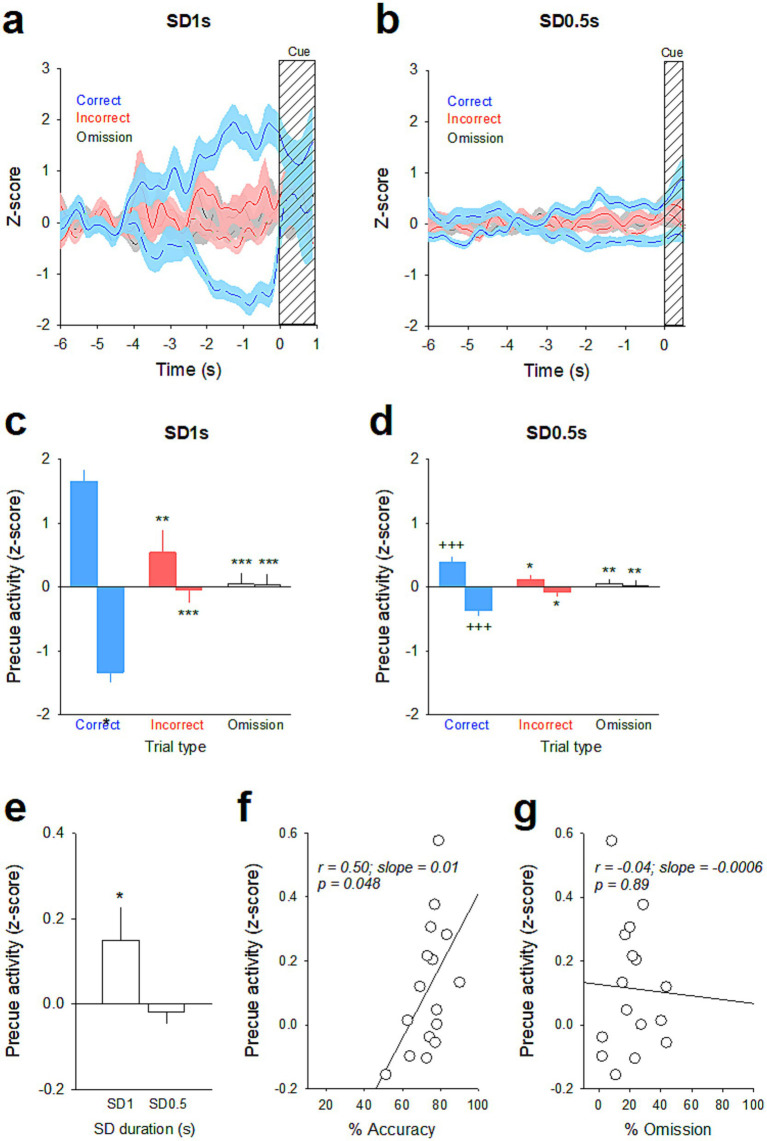
Ventromedial PFC optogenetic inhibition before the SD 1 s and SD 0.5 s cue presentation differentially altered attention. **(a)** Graphic representations of the injections made in the vmPFC (*top*) and of the bilateral illumination of the vmPFC via patch cables (*bottom*). **(b)** Representative images of coronal sections showing the virus expression and fibers implants in the vmPFC (*top*) and immunostaining of eYFP (green) expression in vmPFC pyramidal neurons (red, CAMKII) (*bottom*). **(c)** Representative photomicrographs of cFos labeling in the vmPFC after the opto-inhibition of vmPFC neurons in rats injected with YFP (*top*) or with ArChT (*bottom*). **(d)** Mean (±SEM) density of Fos-positive cells (cFos+ cells/mm^2^) in the vmPFC after the opto-inhibition of the vmPFC in rats injected with YFP (*white bars*) or with ArChT (*green bars*). **p < 0.01, different from YFP. **(e)** Schematic of the location of individual optic fiber tips within the vmPFC. The numbers indicate millimeters anterior to bregma. **(f–i)** Mean (±SEM) percentage of accuracy **(f,h)** and omission **(g,i)** in animals injected either with YFP (*n* = 8) or ArChT3.0 (*n* = 12) under light or no-light conditions during the vmPFC inhibition 2 s before the SD 1 s **(f,g)** or SD 0.5 s **(h,i)** cue presentation. ***p < 0.0 1, different from No Light.

Similarly, the magnitude of precue responses were graded depending on trial type and SD duration (Figure 3). For the SD 1 s, the vmPFC pyramidal subpopulation with increased activity in correct trials displayed lower firing rates in incorrect and omission trials [time × trial type: *F*(118, 5,546) = 1.82; *p* < 0.001; Figure 3a and trial type: *F*(2, 94) = 13.52; *p* < 0.001; Correct vs. Incorrect, *p* = 0.003; Correct vs. Omission, *p* < 0.001 and Incorrect vs. Omission, *p* = 0.30; Figure 3c]. Inversely, the vmPFC pyramidal subpopulation with decreased activity in correct trials was less suppressed in incorrect and omission trials [time × trial type: *F*(118, 5,782) = 2.82; *p* < 0.001; Figure 3a and trial type: *F*(2, 98) = 21.24; *p* < 0.001; Correct vs. Incorrect, *p* < 0.001; Correct vs. Omission, *p* < 0.001 and Incorrect vs. Omission, *p* = 0.95; Figure 3c]. The magnitude of precue responses were also graded at the shorter SD 0.5 s for both the increased [time × trial type: *F*(118, 5,133) = 3.03; *p* < 0.001; Figure 3b and trial type: *F*(2, 87) = 5.94; *p* = 0.004; Correct vs. Incorrect, *p* = 0.02; Correct vs. Omission, *p* = 0.005 and Incorrect vs. Omission, *p* = 0.81; Figure 3d] and the decreased vmPFC pyramidal subpopulations [time × trial type: *F*(118, 4,661) = 2.05; *p* < 0.001; Figure 3b and trial type: *F*(2, 79) = 6.36; *p* = 0.003; Correct vs. Incorrect, *p* = 0.03; Correct vs. Omission, *p* = 0.003 and Incorrect vs. Omission, *p* = 0.67; Figure 3d], but to a lesser extent than in SD 1 s for the correct trials [SD × trial type: *F*(2, 180) = 7.08; *p* < 0.001; SD 1 vs. SD 0.5, *p* < 0.001 and SD × trial type: *F*(2, 177) = 8.29; *p* < 0.001; SD 1 vs. SD 0.5, *p* < 0.001, for the increased and the decreased subpopulations respectively]. Finally, at the population level of all recorded neurons, the neuronal activity during the precue period for all trials combined was significantly higher in SD 1 s than in SD 0.5 s [*F*(1, 122) = 5.45; *p* = 0.015; Figure 3e], and positively correlated with the percentage of accuracy (*r* = 0.50; slope = 0.0113; *p* = 0.048; Figure 3e), but not with the percentage of omission (*r* = −0.04; slope = −0.0006; *p* = 0.89; Figure 3g). Together, these data indicated that changes in attentional load induced changes in attentional performances and associated vmPFC pyramidal precue neuronal activity: the lower the task demand (i.e., SD 1 s), the higher the performance and the higher vmPFC precue firing activity.

### vmPFC opto-inhibition differently affects performances depending on attentional load

**Figure 4:**
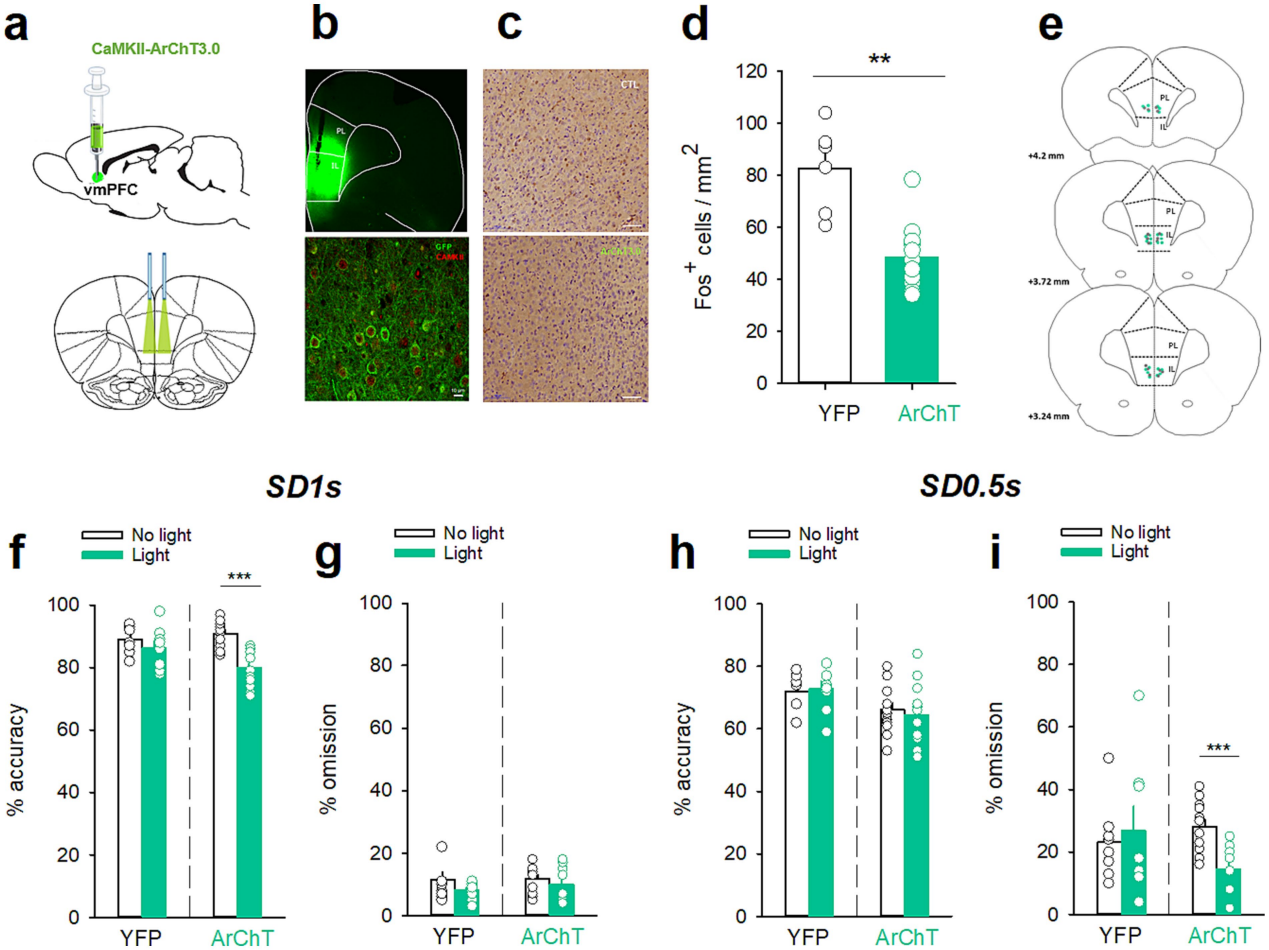
Acute nicotine treatment increases attention. **(a,b)** Mean (±SEM) percentage of accuracy **(a)**, omission **(b)**, and **(c,d)** mean (±SEM) number of correct **(c)** and incorrect **(d)** responses after a saline injection (VEH, *white bars*) and after a nicotine challenge session (NIC, 10 μg/injection, i.v. 1 min before the session, *gray bars*) during the SD 1 s and SD 0.5 s cue presentation. *p < 0.05, different from VEH. ***p < 0.001, different from correct trials.

Based on these observations, we hypothesized that the contribution of vmPFC precue neuronal activity will vary depending on the attentional load and that inhibition of vmPFC neurons should differently impact attention depending on the task difficulty. To test this hypothesis, an adeno-associated virus (AAV) targeting the pyramidal neurons and encoding the archaerhodopsin (CaMKII-ArChT3.0-eYFP; *n* = 12) was bilaterally injected into the vmPFC of a separate group of rats with bilateral implantation of chronic optic fibers in the vmPFC (see Material and methods for details; Figure 4a). As a control, we used a virus expressing YFP only (*n* = 8). The efficacy of this *in vivo* viral strategy was demonstrated by confocal analysis showing that eYFP preferentially colocalized with CaMKII (Figures 4b,e) and indicated efficient transduction in pyramidal neurons (selectivity index >85%). Efficiency of optical stimulation in inhibiting vmPFC activity was demonstrated by cFos immunochemistry showing that light application selectively reduced cFos expression in ArChT but not in YFP rats [*F*(1,16) = 10.76; *p* = 0.005; Figures 4c,d]. At the behavioral level, opto-inhibition of vmPFC pyramidal neurons 2 s prior to cue presentation differentially alters attentional performance depending on the task demand. Consistent with previous studies (Luchicchi et al., 2016), at the long SD 1 s associated with high attentional performances, vmPFC opto-inhibition decreased the accuracy [light × virus interaction: *F*(1,18) = 7.05; *p* = 0.016; ArChT-No light vs. ArChT-Light, *p* < 0.001; Figure 4f] but not the percentage of omission [light × virus interaction: *F*(1,18) = 0.75; *p* = 0.75; Figure 4g]. This effect was due to a decrease in the number of correct responses [light × virus interaction: *F*(1,18) = 6.12; *p* = 0.023; ArChT-No light vs. ArChT-Light, *p* < 0.01; Table 3] and a concomitant increase in the number of incorrect responses [light × virus interaction: *F*(1,18) = 4.90; *p* = 0.041; ArChT-No light vs. ArChT-Light, *p* < 0.001; Table 3]. In contrast, at the shorter SD 0.5 s associated with lower attentional performance, vmPFC opto-inhibition decreased the percentage of omission [light × virus interaction: *F*(1,18) = 13.6; *p* = 0.002; ArChT-No light vs. ArChT-Light, *p* < 0.001; Figure 4i] without affecting the accuracy [light × virus interaction: *F*(1,18) = 0.57; *p* = 0.46; Figure 4h]. This decrease in omission was caused by a greater engagement of the rats in the task as they performed more correct [light × virus interaction: *F*(1,18) = 4.30; *p* = 0.048; ArChT-No light vs. ArChT-Light, *p* < 0.01; Table 3] and incorrect responses [light × virus interaction: *F*(1,18) = 15.68; *p* < 0.001; ArChT-No light vs. ArChT-Light, *p* < 0.001; Table 3], suggesting an increase in general arousal. Optogenetic inhibition of vmPFC pyramidal neurons did not affect the number of premature responses at any SD duration [light × virus interaction: *F*(1,18) = 0.23; *p* = 0.64 and *F*(1,18) = 1.65; *p* = 0.21; for SD 1 s and SD 0.5 s, respectively; Table 3]. Light application also reduced correct [light: *F*(1,18) = 21.9; and *F*(1,18) = 42.0; *p* < 0.001; for SD 1 s and SD 0.5 s, respectively; Table 3] and incorrect latencies [light: *F*(1,18) = 21.9; and *F*(1,18) = 24.5; *p* < 0.001; for SD 1 s and SD 0.5 s, respectively; Table 3] at each SD duration, suggesting that rats may detect the optical stimulation which may act as a cue that accelerates response latency. Importantly however, this decrease in response latencies was observed to a similar extent in both YFP and ArChT rats [correct latency: light × virus interaction: *F*(1,18) = 0.17; *p* = 0.68 and *F*(1,18) = 1.27; *p* = 0.27; *NS*; for SD 1 s and SD 0.5 s, respectively; and incorrect latency: light × virus interaction: *F*(1,18) = 2.23; *p* = 0.15 and *F*(1,18) = 0.68; *p* = 0.42; *NS*; for SD 1 s and SD 0.5 s, respectively; Table 3] and could thus not explain *per se* the changes in performances.

**Table 3:** Effects of optogenetic inhibition of vmPFC pyramidal neurons on 5-CSRTT performances at varying stimulus durations.

	SD 1 s				SD 0.5 s			
	YFP		ArChT		YFP		ArChT	
	No light	Light	No light	Light	No light	Light	No light	Light
Correct responses	77.0 ± 2.5	76.8 ± 2.4	76.2 ± 1.9	67.3 ± 2.4**	44.8 ± 3.2	45.0 ± 5.4	40.6 ± 2.3	50.2 ± 3.6**
Incorrect responses	9.2 ± 1.4	11.4 ± 2.1	7.6 ± 1.0	17.0 ± 1.5***	15.3 ± 1.7	14.4 ± 2.0	20.5 ± 1.2	27.1 ± 2.0***
Premature	4.3 ± 1.5	5.8 ± 1.7	5.1 ± 0.9	6.5 ± 1.3	17.9 ± 2.7	14.4 ± 1.9	14.6 ± 2.2	9.8 ± 1.7
Correct latency (s)^+++^	1.1 ± 0.1	1.0 ± 0.0	1.0 ± 0.0	0.9 ± 0.1	1.2 ± 0.0	1.1 ± 0.1	0.9 ± 0.0	0.8 ± 0.0
Incorrect latency (s)^+++^	1.5 ± 0.1	1.2 ± 0.1	1.5 ± 0.1	0.7 ± 0.1	1.3 ± 0.1	1.0 ± 0.1	1.3 ± 0.1	0.8 ± 0.0
Reward latency (s)	1.3 ± 0.1	1.3 ± 0.1	1.2 ± 0.1	1.2 ± 0.0	1.4 ± 0.1	1.5 ± 0.1	1.3 ± 0.1	1.4 ± 0.1

Data are presented as mean ± SEM. *p < 0.05, **p < 0.01, and ***p < 0.001 significant difference compared to no light. ^+++^*p* < 0.001, global significant effect of light.

### Acute nicotine administration increases attention and potentiates vmPFC neuronal tonic and gamma activities

**Figure 5:**
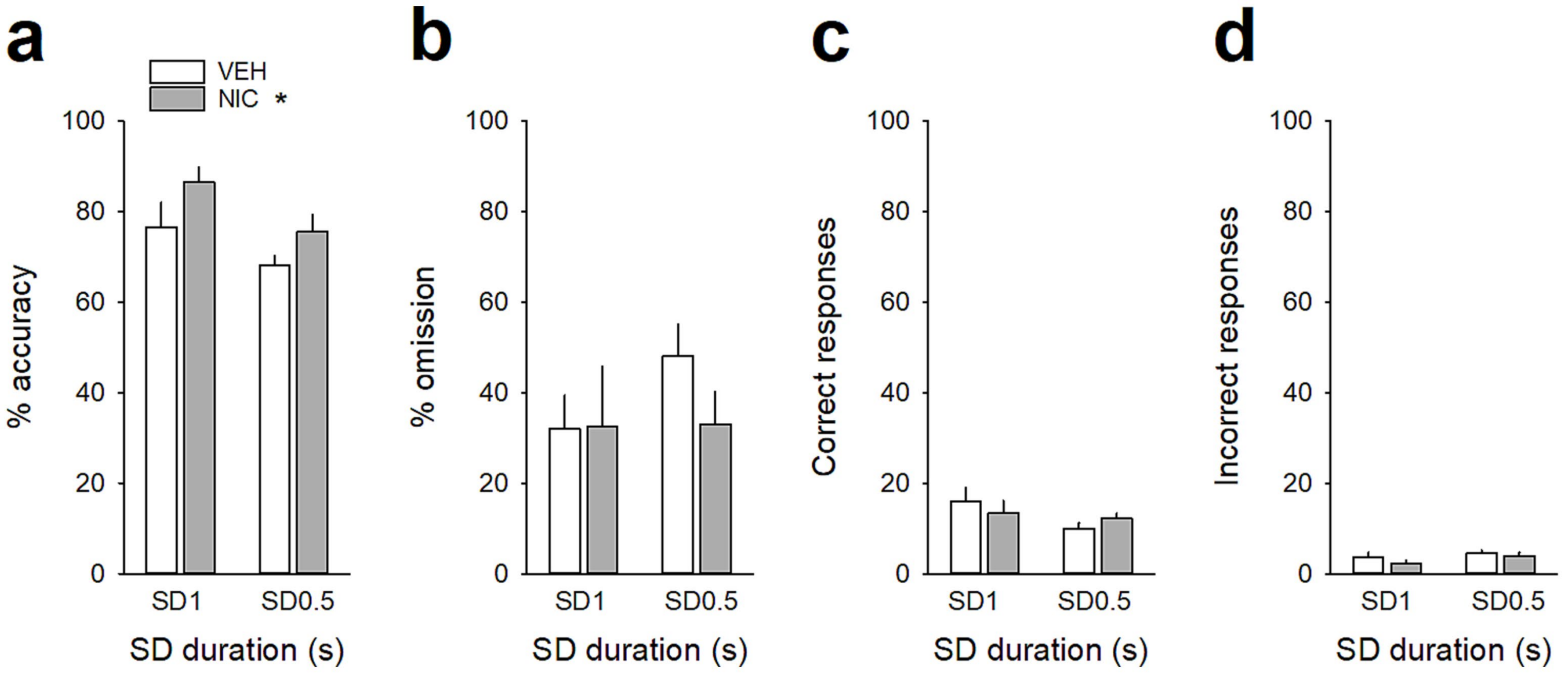
Firing activity of vmPFC precue responsive neurons before the SD 1 s and SD 0.5 s cue presentation. **(a,c)** Mean (±SEM) normalized firing rate (*z*-scores smoothed with a Gaussian filter) of vmPFC precue responses (excitatory, solid line; inhibitory, dashed line) on correct trials (*blue*) and the activity of those same units on incorrect (*red*) and omission (*gray*) trials before the SD 1 s **(a)** and SD 0.5 s **(c)** cue presentation. The lines represent the mean activity and the shaded areas represent the SEM. **(b,d)** Mean magnitude (±SEM) of precue activity (*z*-score) on correct (*blue*), incorrect (*red*) and omission (*gray*) trials before the SD 1 s **(b)** and SD 0.5 s **(d)** cue presentation. *p < 0.05 and **p < 0.01, different from correct trials. ^+++^*p* < 0.001, different from SD 1 s. **(e)** Mean magnitude (±SEM) of precue activity (*z*-score) of all recorded neurons for all trials combined during SD 1 s and SD 0.5 s. *p < 0.05, different from SD 1 s. **(f,g)** The individual precue activity (*z*-score) was positively correlated with individual % of accuracy **(f)** but not with omission **(g)**. ***p < 0.001 Different from correct trials.

**Figure 6:**
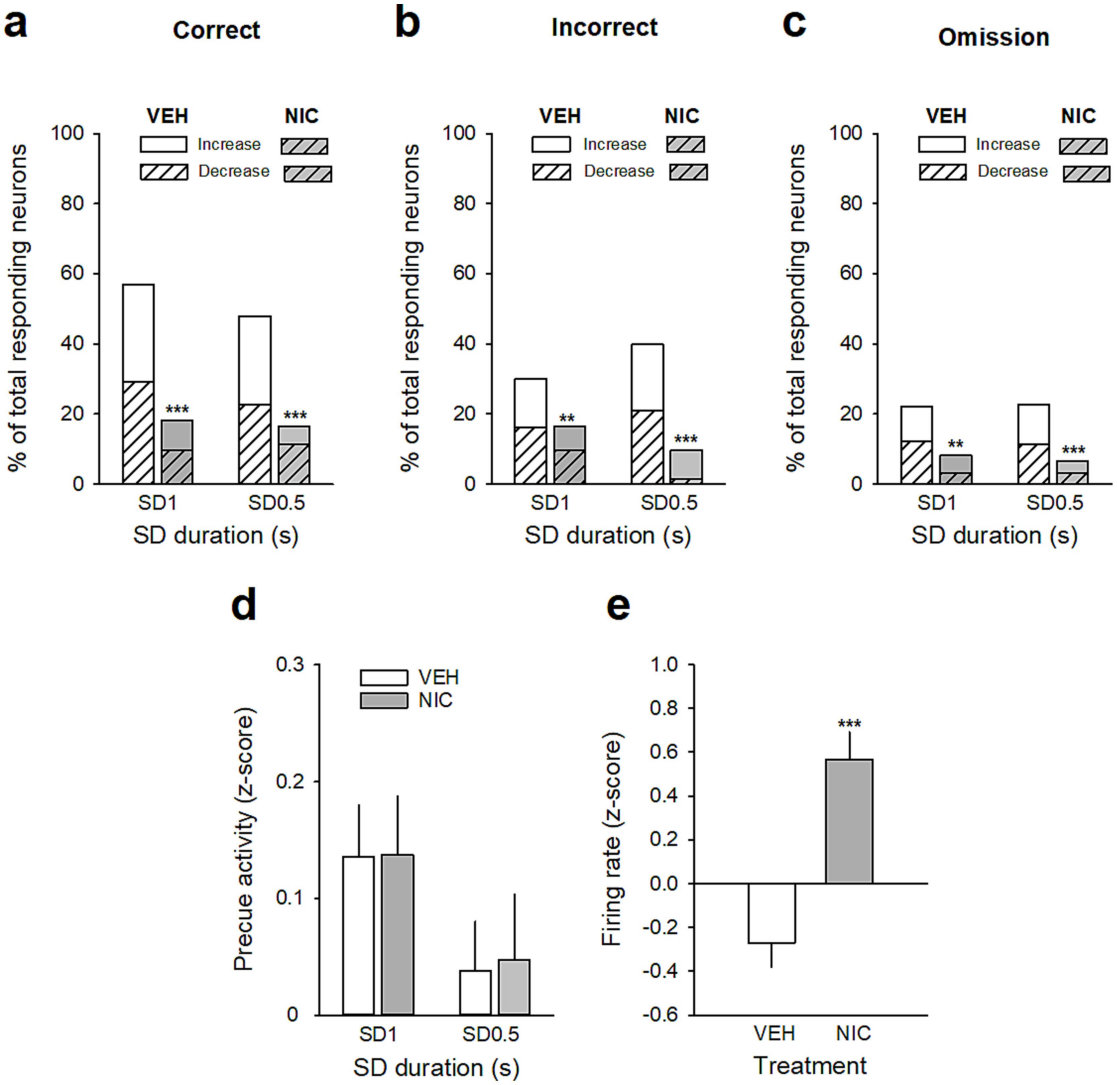
Effects of acute nicotine on vmPFC precue phasic and tonic activities. **(a–c)** Percentage of total neurons responding before the cue on correct **(a)**, incorrect **(b)**, and omission trials **(c)** after a saline injection (VEH, *white bars*) and after a nicotine challenge session (NIC, 10 μg/injection, i.v. 1 min before the session, *gray bars*) during the SD 1 s and SD 0.5 s cue presentation. Excitatory units are shown as solid bars, and inhibitory units are shown as hashed bars. ***p < 0.001, different from BL. **(d)** Mean magnitude (±SEM) of precue activity (*z*-score) of all recorded neurons for all trials combined during SD 1 s and S D0.5 s. **(e)** Mean (±SEM) normalized firing of all recorded neurons (*z*-scores) during the SD variable session after a saline injection (VEH, *white bars*) and after a nicotine challenge session (NIC, 10 μg/injection, i.v. 1 min before the session, *gray bars*). **p < 0.01, different from BL; ***p < 0.001, different from VEH.

**Figure 7:**
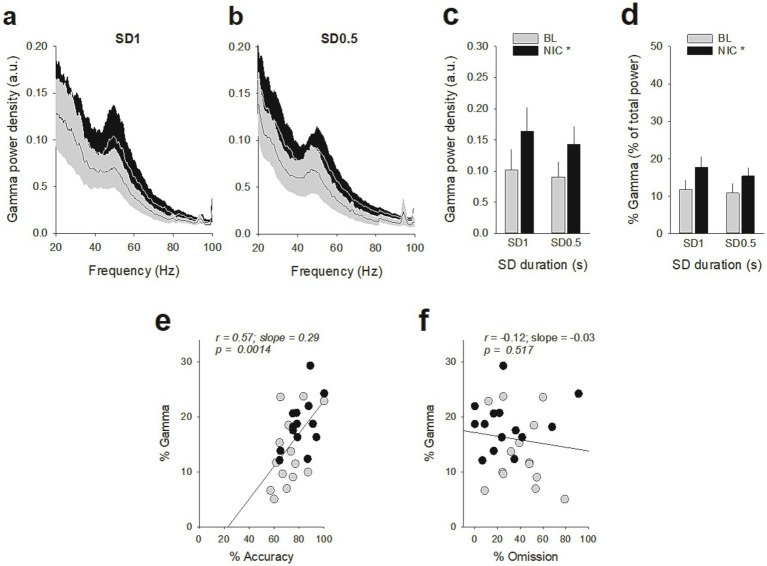
Effects of nicotine on vmPFC gamma oscillations during the SD 1 s and SD 0.5 s cue presentation. **(a,b)** Mean (±SEM) LFP power spectrum density across animals after a saline injection (BL, *gray lines*) and after a nicotine challenge session (NIC, 10 μg/injection, i.v. 1 min before the session, *black lines*) during the SD 1 s **(a)** and SD 0.5 s **(b)**. **(c,d)** Mean (±SEM) gamma power density **(c)** and percentage of gamma oscillations **(d)** after a saline injection (BL, *gray bars*) and after a nicotine challenge session (NIC, 10 μg/injection, i.v. 1 min before the session, *black bars*) during the SD 1 s and SD 0.5 s. *p < 0.05, different from BL. **(e,f)** The individual % of gamma oscillations was highly correlated with individual % of accuracy **(e)** but not with individual % of omission **(f)**.

Because nicotine has well known pro-cognitive properties (Mirza and Stolerman, 1998; Levin et al., 2006), we next assessed the effect of acute non-contingent administration of nicotine (10 μg/inj, i.v., 1 min before the session) on attentional performances and associated vmPFC neuronal activity (Figure 5). As expected, nicotine increased the accuracy at both SD durations [nicotine effect: *F*(1, 6) = 9.90; *p* = 0.019 and nicotine × SD interaction: *F*(1, 6) = 0.05; *p* = 0.83; *NS*; Figure 5a], without affecting the percentage of omission [nicotine effect: *F*(1, 6) = 2.08; *p* = 020; *NS* and nicotine × SD interaction: *F*(1, 6) = 3.40; *p* = 0.11; *NS*; Figure 5b]. Nicotine had no significant effect on any other behavioral measures (Table 4), such as number of premature responses [*F*(1,6) = 0.31; *p* = 0.60], correct response latency [*F*(1,6) = 0.03; *p* = 0.88], incorrect response latency [*F*(1,6) = 0.15; *p* = 0.72], or latency to collect sucrose reward [*F*(1,6) = 0.22; *p* = 0.66]. Surprisingly however, this nicotine treatment almost abolished vmPFC phasic precue responses (Figures 6a–c and Table 5) and had no effect on the precue neuronal activity of all recorded neurons [nicotine effect: *F*(1,60) = 0.009; *p* = 0.92; nicotine × SD interaction: *F*(1,60) = 0.01; *p* = 0.90; *NS*; Figure 6d]. Indeed, the percentage of precue responsive neurons strongly decreased at both SD durations for correct (57% vs. 18%, *Z* = 5.29; *p* < 0.001 and 48% vs. 17%, *Z* = 5.19; *p* < 0.001; for SD 1 s and SD 0.5 s respectively; Figure 6a), incorrect (30% vs. 16%, *Z* = 2.57; *p* < 0.01 and 40% vs. 10%, *Z* = 5.43; *p* < 0.001; for SD 1 s and SD 0.5 s respectively; Figure 6b) and omissions trials (22% vs. 8%, Z = 3.01; *p* < 0.01 and 23% vs. 7%, *Z* = 3.58; *p* < 0.001; for SD 1 s and SD 0.5 s respectively; Figure 6c). This loss of precue phasic responses seems due to an overall basal tonic increase in firing rate of all vmPFC recorded neurons after nicotine injection [*F*(1,53) = 20.21; *p* < 0.001; Figure 6e], an effect that could increase the background firing rate thus limiting the detection of phasic changes in firing and increased in precue neuronal activity (Peoples et al., 2007; Kravitz and Peoples, 2008). In contrast, while increasing the attentional load had no effect on baseline vmPFC gamma power density [SD effect: *F*(1,6) = 1.58; *p* = 0.25; Figures 7a–c] or the percentage of gamma oscillations [SD effect: *F*(1,6) = 0.77; *p* = 0.41; Figure 7d], acute nicotine treatment increased vmPFC gamma power density [nicotine effect: *F*(1,6) = 8.10; *p* = 0.029 5; nicotine × SD interaction: *F*(1,6) = 0.16; *p* = 0.70; Figure 7c] and the percentage of gamma oscillations [nicotine effect: *F*(1,6) = 7.85; *p* = 0.031; nicotine × SD interaction: *F*(1,6) = 0.09; *p* = 0.77; Figure 7d] at both SD durations. Moreover, a correlation analysis revealed that individual percentage of gamma oscillations were highly and positively correlated with individual scores in accuracy (*r* = 0.57; slope = 0.29; *p* = 0.0014; Figure 7e) but not with omissions (*r* = −0.12; slope = −0.03; *p* = 0.52; Figure 7f).

**Table 4:** Effects of acute nicotine (10 μg/kg, i.v.) on 5-CSRTT performances at varying stimulus durations.

	SD 1 s		SD 0.5 s	
	VEH	NIC	VEH	NIC
Premature	2.1 ± 1.2	1.6 ± 0.4	2.0 ± 0.7	2.7 ± 1.3
Correct latency (s)	1.2 ± 0.1	1.0 ± 0.1	1.1 ± 0.2	0.9 ± 0.0
Incorrect latency (s)	1.5 ± 0.3	1.4 ± 0.2	1.2 ± 0.1	1.1 ± 0.2
Reward latency (s)	1.6 ± 0.1	1.6 ± 0.1	1.6 ± 0.1	1.4 ± 0.1

Data are presented as mean ± SEM.

**Table 5:** Effects of acute nicotine (10 μg/kg, i.v.) on the number and percentage of vmPFC neurons responding during the precue period.

		Correct trials		Incorrect trials		Omission trials	
		VEH	NIC	VEH	NIC	VEH	NIC
SD 1 s	Total	70 (57%)	22 (18%)^+++^	37 (30%)***	20 (16%)^++^	27 (22%)***	10 (8%)***^++^
	Increase	34 (28%)	10 (8%)^+++^	17 (14%)**	8 (7%)^+^	12 (10%)***	6 (5%)**
	Decrease	36 (29%)	12 (10%)^+++^	20 (16%)**	12 (10%)	15 (12%)***	4 (3%)**^++^
SD 0.5 s	Total	59 (48%)	20 (16%)^+++^	49 (40%)	12 (10%)^+++^	28 (23%)***	8 (7%)**^+++^
	Increase	31 (25%)	6 (5%)^+++^	23 (19%)	10 (8%)^++^	14 (11%)**	4 (3%)^++^
	Decrease	28 (23%)	14 (12%)^++^	26 (21%)	2 (2%)**^++^_+_	14 (11%)**	4 (3%)*^++^

***p < 0.001 and **p < 0.01, *p < 0.05, significant difference compared to correct trials. ^++^*p* < 0.01 and ^+++^*p* < 0.001, significant difference compared to VEH.

## Discussion

In this study, we tested whether vmPFC neuronal activity is sensitive to changes in attentional 5-CSRTT performances task using two manipulations: a task manipulation increasing task demand by reducing the cue stimulus duration from 1 to 0.5 s, and a pharmacological manipulation administrating an acute nicotine injection (10 μg/inj, i.v.) before the session. As expected from previous research (Mirza and Stolerman, 1998; Hahn et al., 2002; Levin et al., 2006; Amitai and Markou, 2011), increasing task demand led to a decrease in accuracy, while in contrast nicotine injection increased the accuracy. We then recorded and compared vmPFC neuronal activity in these two manipulations that induce opposite effect on attentional performance.

Consistent with previous findings (Totah et al., 2009; Donnelly et al., 2015; Kim et al., 2016), we found that within the vmPFC, a larger proportion of pyramidal neurons showed task engagement with phasic changes in firing rate during the precue period when attention is allocated. These precue responses were observed on each trial (i.e., correct, incorrect or omission trials) and consisted of mixed changes in activity with half of the neurons showing an increase and half of the neurons showing a decrease in firing activity. Importantly, both the proportion of responsive neurons and the magnitude of their responses were graded. That is, fewer units with lower firing activity exhibited phasic responses during the precue period on incorrect and omission trials, compared with correct trials. Thus, vmPFC phasic precue responses appear to be related to the degree of preparatory attention and subsequent response. These precue responses were observed for both SD durations although the number of responsive neurons and the magnitude of precue activity were reduced for the SD 0.5 s along with poorer attentional performances. Moreover, at the population level of all recorded neurons, the neuronal activity during the precue period was globally higher in SD 1 s than in SD 0.5 s, suggesting that enhanced phasic vmPFC activity before the cue presentation facilitates accuracy discrimination.

To further test the link between vmPFC precue activity and attentional performances, we performed optogenetic inhibition of vmPFC pyramidal neurons 2 s before the cue presentation, a time window known to be important for stimulus detection (Luchicchi et al., 2016). Importantly, by doing so in both SD 1 s and SD 0.5 s sessions, we found that vmPFC opto-inhibition had different effects on attentional behavior depending on the SD duration. That is, transient opto-inhibition of vmPFC pyramidal before the SD 1 s cue presentation decreased performance, while the same inhibition protocol before the SD 0.5 s cue presentation increased it. Moreover, these disruptive effects of vmPFC opto-inhibition affected distinct behavioral parameters. Specifically, in SD 1 s, vmPFC opto-inhibition before the cue presentation decreased response accuracy by increasing the number of incorrect responses, while in SD 0.5 s, vmPFC opto-inhibition had no effect on accuracy but increased performance by decreasing the percentage of omissions. This decrease in omission presents as an increase in completed trials, indicating a greater engagement of the rats in the task. Such discrepancies in the effects of optogenetic manipulations of the mPFC in attention have been previously observed in separate studies using different SD cue presentations (Kim et al., 2016; Luchicchi et al., 2016). Importantly, as vmPFC precue activity varied depending on the SD duration, these results suggest that vmPFC opto-inhibition altered performance and behavioral parameters in an activity-dependent manner.

Consistent with previous work (Mirza and Stolerman, 1998; Hahn et al., 2002; Levin et al., 2006), we found that nicotine enhanced attentional performances. Surprisingly however, this nicotine treatment almost abolished vmPFC phasic precue responses, an effect due to an overall increase in tonic firing rate of all vmPFC recorded neurons after nicotine injection that persisted during the entire 5-CSRTT session, and could thus limit the detection of phasic changes in firing (Peoples et al., 2007; Kravitz and Peoples, 2008). Consistent with this, others studies have shown that systemic nicotine increased the tonic firing rate of mPFC pyramidal neurons in rats (Zhang et al., 2012; Bueno-Junior et al., 2017), and the activation of frontal networks during attention tasks in humans (Lawrence et al., 2002). Changes in tonic activity of neuromodulators such as acetylcholine or dopamine are also known to play a role in attention by modulating the general efficacy of cortical information processing (Parikh and Sarter, 2008; Hasselmo and Sarter, 2011; Ellwood et al., 2017; Lohani et al., 2019), an effect that could be responsible for the nicotine-induced tonic increase in vmPFC neuronal activity observed here. Together, these results support the role of persistent tonic changes in vmPFC firing rate as a complementary mechanism by which nicotine may facilitate and strengthen information processing in the PFC.

In addition, while increasing the attentional load had no effect on vmPFC gamma oscillations, nicotine injection increased gamma oscillations and associated attention performances at both SD durations. Thus, in contrast to phasic precue vmPFC neuronal activity, the pharmacological pro-cognitive effect of nicotine on gamma activity are independent of the task demand. Such a strengthening of mPFC gamma power and attention has been previously observed after the administration of nicotine or cholinergic agonists (Pafundo et al., 2013; Bueno-Junior et al., 2017; Howe et al., 2017). Gamma oscillations reflect network synchrony as determined by the balance between excitatory and inhibitory (e.g., E/I balance) activity within a region (Sohal et al., 2009; Uhlhaas and Singer, 2013). Interestingly, optogenetic increase of excitatory neurons in the mPFC has been shown to increase spontaneous gamma power and the E/I balance (Yizhar et al., 2011), suggesting that increases in gamma power could reflect an overall increased activity in cortical circuits (Sohal and Rubenstein, 2019). This is consistent with the concurrent increase in vmPFC tonic neuronal activity and gamma oscillation observed in the present study. Moreover, systemic administration of NMDAR antagonists that preferentially bind and inhibit GABAergic neural activity and enhances glutamatergic neural activity, also increased gamma power oscillations in rats mPFC (Carli et al., 2006; Homayoun and Moghaddam, 2007; Molina et al., 2014). Though we did not measure GABAergic interneurons, our finding that nicotine-induced increase in vmPFC tonic neuronal activity and gamma power supports the view that this pharmacological effect reflects an overall increased level of vmPFC circuit activity rather than of rhythmic neural activity that is synchronized across neurons (Sohal and Rubenstein, 2019). Future studies will be needed to clarify the role of the GABA interneurons in the neuronal mechanisms underlying the pro-cognitive effects of nicotine on vmPFC gamma power.

It is nevertheless surprising that decreasing vmPFC precue activity with optogenetic and decreasing vmPFC precue responsiveness after nicotine injection lead to opposite behavioral changes in performance. One way to solve this apparent paradox is to postulate that, in contrast to gamma oscillations, vmPFC pre-cue activity is simply correlational and does not play a key role in attention. Accordingly then, it is likely that vmPFC opto-inhibition of pyramidal neurons disrupted gamma oscillations in vmPFC which altered attention. Consistent with this, a nicotine injection that almost abolished vmPFC precue responses but increased gamma oscillations also increased accuracy.

Through its diverse anatomical connections, the mPFC is capable of directly influencing a variety of additional regions implicated in attention processing. For instance, neuronal activity in the anterior cingulate cortex (ACC) is increased during a preparatory sustained attentional state in primates and rodents (Johnston et al., 2007; Totah et al., 2009; Totah et al., 2013), and relatively long-lasting chemogenetic inhibition of this area reduced attention-related performance in mice (Koike et al., 2016). Moreover, the mPFC is reciprocally connected with visual areas (Yeterian et al., 2012), and attentional selection appears to be mediated in part by neural synchrony between neurons in the mPFC and visual areas, with phase relationships that seem optimal for increasing the impact of the top-down inputs to the visual cortex (Gregoriou et al., 2009; Bichot et al., 2015; Gregoriou et al., 2015; Siegel et al., 2015). Gamma oscillations in primate PFC and visual cortex are coupled during visual attention, suggesting that PFC gamma entrains sensory regions to attend to appropriate stimuli (Gregoriou et al., 2009; Sreenivasan et al., 2014; Gregoriou et al., 2015). Thus, another possibility is that the pro-attentional effects of nicotine could be attributed to an increase of top-down input to visual system presumably through the enhancement of synchronization in the gamma frequency range.

Together, these results demonstrate a key role of vmPFC neuronal and oscillatory activities in regulating attentional performances when a cue detection is required to produce an adaptive response. Importantly, using two behavioral manipulations (i.e., an increase in the attentional load and a pharmacological intervention), this study reveals the existence of two distinct neuronal processes operating at different timescales and suggests that allocation of attention could be achieved through multiple neuronal mechanisms within the vmPFC.

## Data Availability

The raw data supporting the conclusions of this article will be made available by the authors, without undue reservation.
